# Case Report: Stress Fracture in an International Triple Jumper: Importance of an Integrated Care Approach Which Also Incorporates Biomechanics

**DOI:** 10.3389/fspor.2021.683691

**Published:** 2021-05-28

**Authors:** Edem Allado, Marine Ankri, Frédéric Khiami, Teddy Tamgho, Aghiles Hamroun, Catarina Proenca Lopes, Mathias Poussel, Bruno Chenuel

**Affiliations:** ^1^CHRU-Nancy, University Center of Sports Medicine and Adapted Physical Activity, Nancy, France; ^2^Université de Lorraine, DevAH, Nancy, France; ^3^Department of Orthopedic Surgery and Trauma, Hospital Group (GH) Saint-Louis, Lariboisière, Fernand-Widal, Assistance Publique-Hôpitaux de Paris (APHP), Paris, France; ^4^Department of Orthopedic Surgery and Trauma, Hospital Group (GH) Pitié-Salpêtrière, Charles Foix, Assistance Publique-Hôpitaux de Paris (APHP), Paris, France; ^5^Institut National du Sport, de l'Expertise et de la Performance (INSEP), Paris, France; ^6^Centre for Research in Epidemiology and Population Health, Paris-Saclay University, UMRS 1018, Villejuif, France; ^7^Medicine Department, American Memorial Hospital, Reims, France; ^8^University Hospital of Reims, Reims, France

**Keywords:** stress fracture, biomechanics, elite athlete, triple jump exercise, tibia–injuries

## Abstract

Athletes fear stress fracture (SF) injuries as they can put a premature end to their athletic careers. Understanding any mechanical constraints can suggest preventive management approach. Specifically, for the triple jump, the mechanical stresses that occur during the event appear to be the main factors for risk of injury. This clinical case describes three successive episodes of anterior tibial fracture in an international triple jumper between 2011 and 2013. The first fracture received surgical treatment involving intramedullary nailing. The second fracture occurred in the same location and was considered a recurrence requiring medical treatment, whilst the third was a complete fracture of the surgical material and required surgical revision. These recurrent fractures can be explained by the fatigue of materials (bone and nail) induced by triple jump practice and emphasize the importance of integrating biomechanics into an assessment of the case. The use of biomechanical modelization to identify these weaknesses could be an approach for clinical management of such patients. Observation of the intrinsic mechanical stresses during high-level triple jump may lead to identification of modifiable risk factors for bone fragility.

## Introduction

Stress fracture (SF), also known as fatigue fracture, refers to the notion of fatigue in biomaterial sciences. SFs are particularly common in athletes, with a prevalence of 10% in this specific population, compared to 0.9–6.9% in the general population, highlighting the consequence of physical overwork in athletes (Crossley et al., 1999; Robertson and Wood, 2015; Sanchez-Santos et al., 2017). Approximately 80–95% of SFs affect the lower limbs, 23–73% of which involve the tibia (Iwamoto and Takeda, 2003; Kahanov et al., 2015; Robertson and Wood, 2015). SFs predominantly occur in people who perform difficult and/or weight-bearing movements, such as runners, or in some specific professions, such as the military (Jones, 2002). Athletes fear SF injuries since these could prematurely end their careers. The high-level sports context therefore highlights the need for surgical management in this particular group compared to the medical treatment (mainly simple rest) which is commonly recommended for the general population.

SF risk factors include environment (alterable), technical movements, equipment, hard or rough terrain, overtraining, and abnormal morphological characteristics, such as thinness, large size, low muscle mass, and age >23–25 years old (Marcelli and Lafage-Proust, 2000; Iwamoto and Takeda, 2003; Prouteau et al., 2005; Varner et al., 2005; Kahanov et al., 2015; Hadid et al., 2018). Local factors such as unequal lengths of the lower limbs have also been identified as risk factors. The management of modifiable risk factors is therefore essential.

In athletics, cross country disciplines are particularly affected by fatigue fractures (Crossley et al., 1999; Robertson and Wood, 2015; Sanchez-Santos et al., 2017). However, this type of injury is also found in jumping events such as the triple jump. In this clinical case, we observed recurrent fatigue fractures in an area where traumatic injuries would be more expected, especially at an elite level. Understanding the physical mechanism underlying such an injury was important to improve the rehabilitation process.

Here we describe the unusual case of a top-level triple jumper who experienced three SFs involving the same uncommon region (before and after surgery) and which occurred over a short period of time (30 months). We also describe the contribution of biomechanics in the efficient care program for rehabilitation. The patient approved this submission for publication and gave consent for the use of his data and photographs.

## Case Report

**Figure 1:**
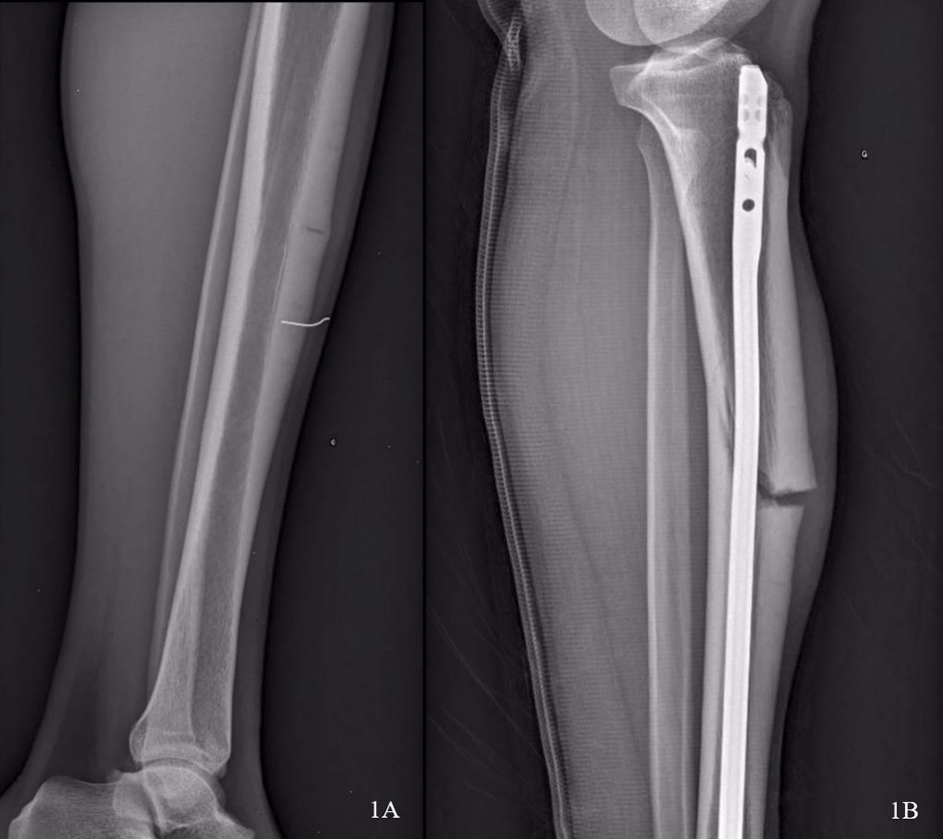
Conventional radiography (profile) of the left limb showing the anterior tibial stress fracture with significant cortical thickening **(A)** and the complete anterior tibial fracture on pseudarthrosis with deformation of the intramedullary nail **(B)**.

An elite triple jumper (internationally, one of the top 10 in this field), free from any previous bone injury, experienced his first episode of pain from the anterior cortex of the left tibia in July 2011 at the age of 22. The pain forced him to end his participation in an international contest during the final three jumps. After these first symptoms, the athlete was able to continue his training for a few weeks before having to suspend practice due to evidence of an incomplete fracture of the anterior tibial cortex (see Figure 1A). Surgical management was initiated with decortication, bone grafting and intramedullary nailing. Partial and full weight-bearing activities were allowed after 48 h and on day 15 (D15) after surgery, respectively. The rehabilitation was done in a center specialized in the rehabilitation of high-level athletes (D15–D45). During this period, the patient benefited from a progressive support and strength training. The patient returned to training sessions with physiotherapist (D45–D60), started to run again on D60, and was able to perform his first jumps on D90. The athlete was able to return to triple jump 6 months after surgery.

In October 2012, patient weighed 83 kg for 1.87 cm during training. In the context of recurrent pain symptoms on the anterior side of the left leg, the diagnosis of pseudarthrosis with no surgical indication was made thanks to conventional radiography. Medical management was initiated, allowing pain control and the regression of radiographic abnormalities. Specifically, treatment comprised rest combined with vitamin D supplementation; indeed, the patient's blood test revealed vitamin D deficiency, with a serum level <50 mmol/L (normal range is 75–250 mmol/L). He had a poor vegetable diet, mainly based on white meat and starches.

Regarding physical work out, the number of training sessions per week for the athlete was 6–9 (2-h sessions) including two sessions per week of bodybuilding (1 type over 70% maximal load//1 < 70%). He also performed around 300 jumps per week from September to December and from March to the end of May and 6–20 jumps per week from January to February and from June to July/August (competition phase). In competition, the elite athlete had a run approach speed of 10.8–11.2 m/s. The means distances of triple jump phase (hop, step, and jump) were 6.10–6.20, 5.10–5.20, and 6.10–6.20 m, respectively.

After his return to international competition level (i.e., jumps beyond 17.50 m), the athlete experienced a new episode of pain in the same fracture area in November 2013. Once again, pain occurred during ground impact when completing the last jump. Conventional radiography performed on the trauma day showed a complete fracture of the tibia with a deformation of the intramedullary nail (Figure 1B).

The following day, the surgical equipment was removed, and a new intramedullary nail was set up. Management of the pseudarthrosis fracture was performed, consisting of decortication associated with a bone autograft taken from the iliac crest (cancellous bone graft), and installation of a neutralization plate.

Full weight-bearing activities were authorized on D30 and running on D90. The athlete resumed jumping 5 months after surgery.

The post-surgical rehabilitation phase began with 1 month of immobilization and 2 months of motor physiotherapy. The return-to-field phase required a complete understanding of the athlete to study the mechanism of injury and, to this end, a precise kinematic analysis of the jumps was performed to identify modifiable biomechanical errors.

**Figure 2:**
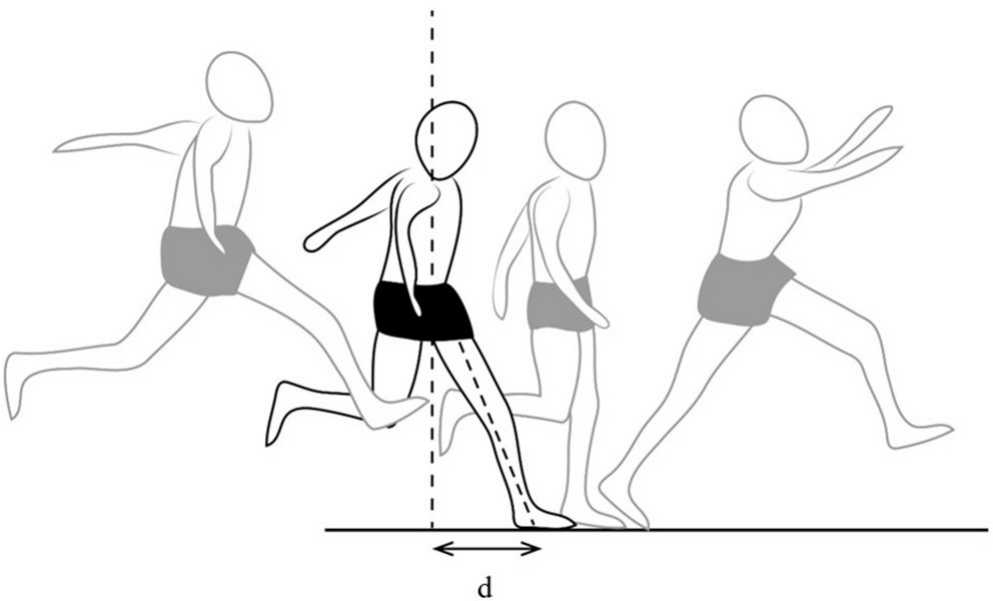
Foot position and pelvis projection during jump receptions in triple jump. “d” is a distance between foot position and pelvis.

Immediately after the run-up, the goal of the athlete was to maintain his maximum speed at every ground impact, applying the same model as “the ricochet.” To do this, the extensor muscles of the foot, leg and hip fight against the sharpening induced by every reception to avoid a speed decrease. The interrelation between foot and pelvis positions during these receptions is one of the factors favoring jumping-related stress. When the foot is in a very anterior position compared to the line drawn by the pelvis and the shoulder, the effort appears to be greater for the quadriceps (and for the sartorius to a certain extent as well), which is the only extensor group located on the front side of the leg (Figure 2). This is how this muscular group, inserted directly or indirectly on the anterior surface of the tibia, can induce repeated microtraumas, thus subjecting the cortical bone to fragilization.

The video analysis of the gestures of the triple jumper allowed us to identify this type of positioning. In consequence, we tried to focus on diminishing the anteriorization of the foot at reception (distance “d”—Figure 2), so as to transfer the shock absorption to the other extensor groups, located on the posterior face. This gesture correction required specific work, based on three modalities:

- Material: the promotion of a damping process by a better absorption of the impact forces. The training track cover was adapted, and we used personalized damping heels in the athlete's shoes.
- Physical: several repetitions of the gesture with the intent to correct it on low kinetic jumps (reduced run-up) during training sessions. The aim of this approach was to allow a better gesture appropriation.
- Mental: a psychological stimulation through mental visualization of the different sensations perceived by the high-level athlete. This phase is essential to potentiate the rapid assimilation of the corrected movement in competition. Indeed, this work allows the stimulation of proprioception and an improved synchronization of the involved muscles (Jeannerod, 2001; Malouin and Richards, 2010; Rulleau and Toussaint, 2014; Keilani et al., 2016).

One year later, the athlete returned to international competitions, where he performed jumps beyond the international marker level of 17.50 m and, since then, there has been no recurrence of pain. He did not show any tibia injury until over the end of his career. Unfortunately, he experienced a left Achilles tendon rupture in 2015 and a f right medial knee condyle fracture related to a benign tumor in 2016.

## Discussion

Through this original clinical case, we describe an uncommon recurrent fatigue fracture on the anterior face of the tibia, occurring in an international level athlete. This allowed us to focus on the biomechanical contribution to the construction of a personalized rehabilitation program. In addition, this observation gave us the opportunity to explore the specificities of a discipline that is not well-studied.

SFs frequently occur in elite athletes. In our case report, the recurrence of a fracture 30 months after the athlete had returned to his initial level of performance can be mostly explained by the presence of pseudarthrosis in 2012. Studies find a time recovery physical activity between 3 and 10 months in 90% of cases (Varner et al., 2005; Borens et al., 2006; Miyamoto et al., 2009; Cruz et al., 2013; Robertson and Wood, 2015). The main factors influencing the recovery are the absence of pain, the athletic level, and the fear of a new injury.

The latter resulted in a weakness of the structures around the initial intramedullary nail, which experienced material fatigue under the strong mechanical constraints.

The triple jump conditions can partly explain the recurrent episodes of fracture (Hadid et al., 2018). Indeed, this activity requires unusual physical qualities of resistance to repetitive ground impacts, where the weight of the athlete may reach 7–12 times its resting value (Ramey and Williams, 1985; Perttunen et al., 2000). Under repeated stresses, a material usually undergoes three steps of deformation: elastic deformation, reversible deformation induced by “low” stress, and non-reversible plastic deformation induced by “high” stress. Finally, beyond a certain threshold, the material enters a complete rupture zone. Although bone is a living organ, repeated stresses below the rupture threshold can induce a true fracture when bone reconstruction phenomena (driven by osteoblasts) are overcome by resorption phenomena (related to osteoclast activity). The Wöhler curve representing stress as a function of the cycle number permits estimation of the degree of damage related to material fatigue (Carter and Caler, 1985). The triple jump is a low cycle test (200–300 weekly bursts) with high unit stress amplitude. Thus, for the same number of impacts (cycles), a significant stress may approach the yield point, and the bone may then twist.

Physiological and pathological studies provide already pertinent data for description, modelization and hypothesis elaboration but the use of modelization derived from material science concepts could provide an understanding of the specific biomechanical pathophysiology involved in clinical cases.

In addition, to prevent recurrence, risk factor management was addressed through the multiple approaches of remodeling the training program by decreasing the number of jumps, adaptation of shoes for better impact management and correction of vitamin D deficiency (Kahanov et al., 2015; Miller and Best, 2016; Sanchez-Santos et al., 2017).

This case report illustrates how the perception of the gesture requires exploration in athletic disciplines, most particularly in triple jump. This perception may be influenced by a state of intrinsic (illness) and/or extrinsic fatigue (over-training) thus inducing a technical error with important consequences. For proper treatment of elite athlete injuries, an appropriate care program should integrate several approaches, such as gesture analysis (with biomechanics and cinematic contributions), ergotherapy and psychological work, in order to adapt management, training work and eventually rehabilitation to avoid injury and its recurrence.

## Subject Perspective and Conclusion

This clinical case of recurrent SF in an international triple jumper highlights the necessity of a global approach to the analysis of an athlete's movements, mechanical stress and the environment, and in the additional use of concepts derived from biomaterial sciences.

Early surgical management is generally discussed for international elite athletes, as early return to competition is a key issue. There is now evidence that pseudarthrosis and/or recurrent injuries following delayed treatment could prematurely end an athletic career. Nevertheless, the iatrogenic risk of surgical intervention must be considered in the benefit/risk balance assessment.

An integrated approach will permit a more precise diagnosis of the underlying injury mechanism and the initiation of a personalized and adapted care program. In addition to the standard recommendations and complementary medical advice, biomechanical modelization could be an interesting tool to incorporate into an integrated care approach in this population of athletes.

This case report illustrates the advantage of using a comprehensive approach for the management of elite athlete recovery.

## Data Availability Statement

The original contributions presented in the study are included in the article/supplementary material, further inquiries can be directed to the corresponding author.

## Ethics Statement

Ethical review and approval was not required for the study on human participants in accordance with the local legislation and institutional requirements. Written informed consent to participate in this study was provided by the participants' legal guardian/next of kin. The patient approved the submission for publication of his data and photographs.

## Author Contributions

EA made substantial contributions to conception, drafting the article, and critical revision of the article for important intellectual content. MA and FK collected the data and made substantial contributions to the critical revision of the article. TT, AH, CP, and MP made substantial contributions to the critical revision of the article. BC made substantial contributions to the design of the work and to critical revision of the article. All authors have read and approved the final version of the manuscript and agree with the order of presentation of the authors.

## Conflict of Interest

The authors declare that the research was conducted in the absence of any commercial or financial relationships that could be construed as a potential conflict of interest.
